# Migration Behavior of Lubricants in Polypropylene Composites under Accelerated Thermal Aging

**DOI:** 10.3390/polym13111723

**Published:** 2021-05-25

**Authors:** Mun-Gyu Bak, Jong-Sung Won, Seon-Woong Koo, Arom Oh, Han-Ki Lee, Dae-Sik Kim, Seung-Goo Lee

**Affiliations:** 1 Department of Plastic Materials Research Team, Automotive Research & Development, Hyundai Motor Group, Hwaseong-si 445-010, Korea; mg_bak@hyundai.com (M.-G.B.); arom@hyundai.com (A.O.); HK_Lee@hyundai.com (H.-K.L.); kimds@hyundai.com (D.-S.K.); 2 Robert Frederick Smith School of Chemical and Biomolecular Engineering, Cornell University, Ithaca, NY 14853, USA; jw2636@cornell.edu; 3 Department of Advanced Organic Materials & Textile Engineering, Chungnam National University, Daejeon 34134, Korea; muzicle@torayamk.com

**Keywords:** polypropylene, migration, lubricant, accelerated aging, additives, thermoplastic composites, polymer interface

## Abstract

The surface migration of lubricants degrades the quality of thermoplastic polymer composites. In this study, the surface migration of lubricants in polypropylene composites were studied to improve the quality of the composites. Polypropylene (PP)/lubricant composites were manufactured using a co-rotating twin-screw extruder and injection molding, and the migration phenomena of the lubricant in the PP/lubricant composites were investigated under accelerated aging conditions with temperatures in the range of 20 to 90 °C and humidity of 100% for 72 h. The interrelation between the surface migration properties of PP/lubricant composites were investigated by considering their microstructural and morphological features, which were influenced by the thermal aging conditions. Further, the microstructural and morphological features were examined by contact angle, surface energy, attenuated total reflectance Fourier-transform infrared spectrometry, X-ray photoelectron spectroscopy, close-up digital imaging, and atomic force microscopy analyses. The polypropylene composites containing the magnesium stearate as the lubricant were found to exhibit a more stable migration behavior than the polypropylene composites containing a calcium stearate lubricant. This is attributed to multiple synergistic factors, such as interfacial tension and work of adhesion between PP and the lubricant. The findings of this study can be utilized to effectively manufacture high-quality thermoplastic composites for the fourth industrial revolution.

## 1. Introduction

Thermoplastic polymer products have become indispensable in everyday life, as they are essential components of clothing, building, transportation, and communication materials. Thermoplastic polymers are of great interest to the industry and academia owing to their advantageous properties over thermosetting polymers, such as recyclability, low cost, lightweight, design flexibility, strategic and selective reinforcement capability, and short total process cycle time. One of the most widely used and well-known thermoplastics is polypropylene (PP) [1,2,3,4,5]. However, PP has low moisture absorption, high electrical insulation, and poor mechanical properties. Therefore, PP is generally incorporated with lubricants such as antistatic agents, slip agents, UV stabilizers, and antioxidants, in order to improve its processability and physical and chemical properties, as well as prevent discoloration and degradation of mechanical properties by oxidation. Most of these lubricants are low-molecular-weight compounds and are added in small amounts (less than 3% in most cases). These lubricants must be incorporated well into the polymer to ensure their intended function [6].

It has been found that the surface migration of these lubricants causes quality deterioration, especially in initial quality studies and vehicle dependability studies. The International Union of Pure and Applied Chemistry defined migration as a process in which one component of a polymer mixture, usually not a polymer, undergoes phase separation and migration to the external surface of the mixture [7,8]. This migration phenomenon can modify the surface properties required for specific applications, such as car interior and food packaging materials. In particular, the migration of lubricants and antistatic agents not only changes the appearance of the final product but also reduces the antistatic properties of PP. These undesirable changes in the PP surface composition lead to major problems in terms of mechanical failure, quality-control rejection due to surface contamination, and surface inhomogeneity [9,10,11]. Therefore, additive characterization at the PP surface is necessary to improve manufacturing processes in order to increase the quality and competitiveness of their products. Hence, studies are being conducted to develop methods to confirm the surface migration of lubricants in PP/lubricant composites [12,13]. Furthermore, some lubricants are known to be potentially carcinogenic to humans and are endocrine disruptors. Therefore, assessing a consumer’s exposure to lubricants originating from polymer materials is very important [14].

In this study, in order to investigate the phenomenon that causes the quality deterioration of polypropylene composites, the work of adhesion between PP and lubricants was derived and the surface migration phenomenon was investigated. PP/lubricant composites were first manufactured using a co-rotating twin-screw extruder, and then aging tests were carried out to investigate the migration phenomena between PP and the lubricant. To this end, PP specimens were fabricated by injection molding, and accelerated aging tests were conducted on PP/lubricant composites at different thermal aging temperatures of 20 to 90 °C and humidity of 100% for 72 h. The microstructural and morphological features of the PP/lubricant composites were characterized by evaluating the contact angles, surface free energies, interfacial tensions, and work of adhesion values between PP and the lubricant. The surface chemical compositions of the PP/lubricant composites were measured, and the results were discussed considering the interactions and structural features.

## 2. Materials and Methods

### 2.1. Materials

We used PP chips with a melt flow index of 30 g/10 min and molecular weight of 229,300 g/mol (Bx3800, Sk Chemicals, Seongnam, Korea) as the polymer matrix. Mg (Magnesium salt of stearic acid, particle size of 10 um) and Ca (Calcium salt of stearic acid, particle size of 20 um) in powder form were incorporated into PP as lubricants (SD Korea, Hwaseong, Korea).

### 2.2. Preparation of PP/Lubricant Composites

**Figure 1:**
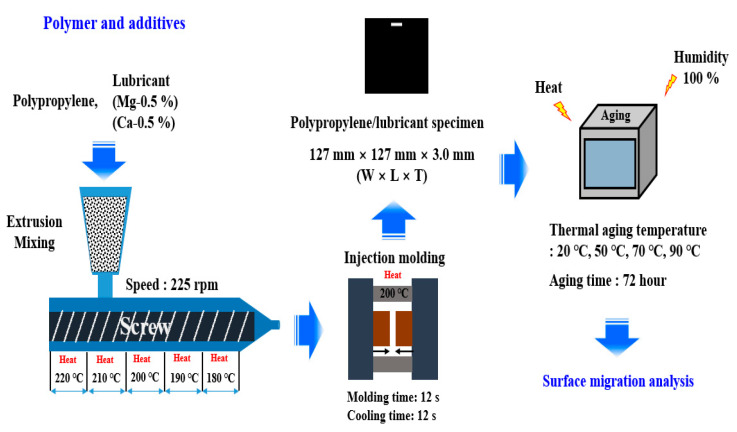
Schematic of procedure followed for the surface migration analysis of Polypropylene/lubricant composites by thermal and humidity aging.

Figure 1 shows a schematic of the preparation procedure used for the surface-migration analysis of PP/lubricant composites subjected to thermal and humidity aging. Prior to extrusion, PP and the lubricants were dried in an oven for 3 h at 70 °C. The PP/lubricant composites were extruded using a co-rotating twin-screw extruder (TEK20, S.M Platek, Republic of Korea) with a L/D ratio of 42 and an intermeshing screw configuration. The screw speed was set to 225 rpm, and the processing temperature was set in the range of 180–220 °C. PP/lubricant composites were prepared by mixing master batches with lubricants (Mg or Ca) at loadings of 0.5 wt.%. The lubricant loading was selected as 0.5 wt.%, which is widely used in actual product development for automobile interior materials. The extrudates were pelletized using an S.M. Platek pelletizer. Later, PP/lubricant composite specimens for testing were prepared using injection molding (IDE140ENII, Goldstar, Seoul, Korea). Specimens for the aging test were manufactured with dimensions of 127 mm × 127 mm × 3.0 mm. The injection molding conditions included an injection temperature of 200 °C, molding time of 12 s, cooling time of 12 s, and mold opening time of 10 s. Subsequently, accelerated thermal and humidity aging was carried out in a thermo-hygrostat chamber at different temperatures (20–90 °C) and humidity of 100% for 72 h. Finally, the surface migration phenomena of the PP/lubricant composites were analyzed under accelerated aging conditions.

### 2.3. Characterization of PP/Lubricant Composites

A Fourier-transform infrared (FTIR) spectrometer (Bruker Optic GmbH, ALPHA-P) equipped with an attenuated total reflectance (ATR) accessory was used to examine the surface composition of the PP/lubricant nanocomposites. The spectra were recorded in the transmission mode in the range of 4000–500 cm^−1^. The spectral resolution was 4 cm^-1^, and 128 scans were accumulated at a high signal-to-noise ratio. Further, the surface chemical compositions of the PP/lubricant composites were analyzed using X-ray photoelectron spectrometry (XPS, Multilab 2000, Thermo Fisher Scientific, Waltham, MA, USA). The XPS spectra were obtained using Al Kα (hv = 1400 eV) radiation as the monochromatic X-ray source at an operating voltage of 12 kV.

The surface morphological features of the PP/lubricant composites were characterized using a digital camera (IFS-28, Canon, Tokyo, Japan). Additionally, atomic force microscopy (AFM, Bioscope, Digital Instruments, Tonawanda, NY, USA) was utilized to observe the three-dimensional topographic images of the PP/lubricant composites and evaluate their root mean square (RMS) roughness.

The contact angles of water droplets coming into contact with the samples were measured using a DSA25 Drop Shape Analyzer System (KRUSS, Hamburg, Germany). Deionized water and diiodomethane were used as probe liquids for hydrophilic and hydrophobic interactions, respectively; five independent measurements were averaged.

### 2.4. Characterization of PP/Lubricant Composite Surface

#### 2.4.1. Sessile Drop Method

The sessile drop method is an optical analytical method based on contact angle measurements and can be used to evaluate the wettability of a solid surface. While the surface tension or surface free energy of a liquid can be measured directly using a tensiometer, it is very difficult to directly measure the surface free energy of a solid. Fowkes, Good, and van Oss developed a method to measure the surface free energy of liquids and solids [15].

Owens and Wendt continued to develop the Fowkes idea and extended it to polar components. Using this method, the free energy *γ_sl_* of a liquid/solid interface can be expressed as follows [16]:

(1) $$\gamma_{sl}=\gamma_{s}+\gamma_{l}-2\sqrt{\gamma_{s}^{d}\gamma_{l}^{d}}-2\sqrt{\gamma_{s}^{p}\gamma_{l}^{p}}$$

where *γ_l_* is the surface free energy of the liquid, *γ_s_* is the surface free energy of the solid, *γ_l_^d^* is the dispersive component of liquid-surface free energy, *γ_l_^p^* is the polar component of liquid-surface free energy, *γ_s_^d^* is the dispersive component of solid-surface free energy, and *γ_s_^p^* is the polar component of solid-surface free energy. According to Fowkes, the surface free energy of a solid can be calculated using the dispersive component and the contact angle as follows:

(2) $$\frac{1+cos\theta }{2}\frac{\gamma_{l}}{\sqrt{\gamma_{l}^{d}}}=\sqrt{\gamma_{s}^{p}\;}\;\times \;\sqrt{\frac{\gamma_{l}^{p}}{\gamma_{l}^{d}}}+\sqrt{\gamma_{s}^{d}}.$$

The dispersive component *γ_s_^d^* and polar component *γ_s_^p^* can be obtained by analyzing Equation (1) linearly. The surface free energies and components of the blended reagents are listed in Table 1.

#### 2.4.2. Column Wicking Method

**Figure 2:**
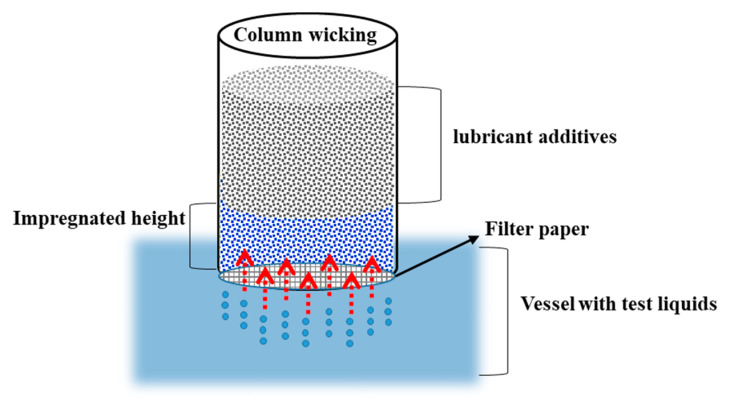
Schematic diagram showing column wicking of lubricant.

A liquid transfer pipe with a 10 mm diameter and a 10 cm length was used (Figure 2). The lubricant was placed in the liquid transfer pipe at a certain height, and a slight concussion was performed to evenly distribute the sample before it was placed in the impregnating liquid. The positions reached by the impregnating liquid were marked every 10 s and were subsequently measured using Vernier calipers. Finally, the relationship between the dipping time and the impregnation distance was obtained. All the experiments were conducted at room temperature; the test values presented are averages of five individual experimental results.

The surface free energy of the lubricant and its components were obtained using the column wicking method, which is based on the Washburn theory (Equation (3)) and the van Oss-Chaudhury-Good portfolio theory (Equation (4)):

(3) $$\frac{X^{2}}{t}=\frac{\gamma_{L}Rcos\theta }{2\eta }.$$

In Equation (3), *X* is the impregnated distance, *t* is the dipping time, *γ_L_* is the surface tension, *R* is the capillary effective radius, *θ* is the contact angle between the liquid and solid, and *η* is the liquid viscosity:

(4) $$\frac{\gamma_{L}\left(1+cos\theta \right)}{2}=(\gamma_{S}^{LW}\gamma_{L}^{LW}{)}^{1/2}+(\gamma_{S}^{+}\gamma_{L}^{-}{)}^{1/2}+(\gamma_{S}^{-}\gamma_{L}^{+}{)}^{1/2}.$$

In Equation (4), *γ_L_* is the liquid surface tension or solid surface energy, and *θ* is the contact angle between the liquid and solid; the subscripts *S* and *L* indicate the solid and liquid, respectively; *γ_S_^LW^* and *γ_L_^LW^* are the solid and liquid Lifshitz-van der Waals force, respectively; *γ_S_*^+^ and *γ_L_*^+^ are the solid and liquid Lewis acid force, respectively; *γ_S_*^−^ and *γ_L_*^−^ are the solid and liquid Lewis alkali force, respectively.

In Equation (3), when a low surface-energy liquid is used, the liquid fully infiltrates the lubricant, so cos *θ* = 1, which results in:

(5) $$\frac{X^{2}}{t}=\frac{\gamma_{L}R}{2\eta }$$

where *γ_L_*^+^ and *γ_L_*^−^ of a test liquid are both zero, Equation (4) can be rewritten as follows:

(6) $$\frac{\gamma_{L}\left(1+cos\theta \right)}{2}=(\gamma_{S}^{LW}\gamma_{L}^{LW}{)}^{1/2}$$

The capillary effective radius (R) can be obtained using a liquid with a low surface energy according to Equation (5) (n-hexane was used in this study). Diiodomethane was used to calculate the value of cos *θ* using the relationship between *R* and *X*^2^/*t*, where *X* is the penetrated distance and *t* is time. Subsequently, the *γ_S_^LW^* values of the lubricant was obtained using Equation (6). To calculate the Lewis acid force *γ_S_*^+^ and the Lewis alkali force *γ_S_***^–^** using the van Oss–Chaudhury–Good theory, toluene and chloroform with specific surface free energy components were needed. The Lewis acid-alkali force *γ_S_^AB^* of the lubricant was calculated using the equation *γ_S_^AB^* = 2(*γ_S_^+^ γ_S_^−^*)^1/2^. The total surface energy *γ_S_^TOT^* consists of *γ_S_^LW^* and *γ_S_^AB^*.

The surface free energies, Lifshitz-van der Waals force, Lewis acid force, Lewis alkali force, and viscosities of the test liquids (distilled water, diiodomethane, formamide, toluene, and chloroform) are listed in Table 2.

## 3. Results and Discussion

### 3.1. Contact Angle and Surface Free Energy of PP/Lubricant Composites

**Figure 3:**
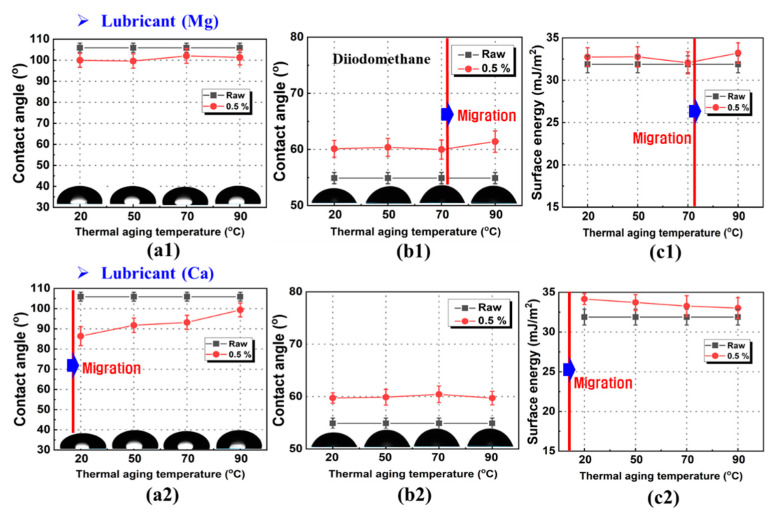
Variation in contact angles and surface energies of Polypropylene/lubricant composites aged at different temperatures at a humidity of 100% relative to different test liquids: (**a**) Distilled water, (**b**) diiodomethane, and (**c**) surface energy.

The contact angle is a measure of the wettability and surface roughening of a polymer surface. Therefore, the extent of surface modification due to accelerated aging can be evaluated using contact angle measurements. Figure 3 shows the variations in the contact angle and surface energy of the test liquids and PP/lubricant nanocomposites aged under different thermal conditions and 100% humidity. Before the aging treatment, the surfaces of the PP/lubricant composites exhibited contact angles of 105.9° and 54.9° with distilled water and diiodomethane, respectively. After accelerated aging, the contact angle decreased as the temperature increased. The variation in the hydrophilicity of the aged samples is illustrated in Figure 3a1,a2. Different trends were observed for the two PP/lubricant composites. With the Ca lubricant, the surface changed rapidly at the accelerated aging temperature of 20 °C, but the Mg lubricant did not change even at 90 °C.

These results suggest that the bonding strength between PP and Mg is higher than that between PP and Ca. The hydrophobicity of the PP/lubricant composite surfaces after 72 h of thermal aging at 20–90 °C was assessed using diiodomethane contact angle measurements. The measurements of the contact angle at different temperatures during thermal aging confirmed that the aged surfaces were unstable, and the hydrophobicity increased with the thermal aging temperature. A large change was observed in the contact angle of the specimen containing Mg lubricant subjected to aging treatment at high temperatures (Figure 3b1,b2). Indeed, in this case, it is assumed that the lubricant evaporated as the aging temperature increased, and hence, migrated to the surface of the PP. As the thermal aging temperature increased, the lubricant evaporated on the surface, and thus, the contact angle decreased. It was found that Ca has lower contact angles than Mg at an aging temperature of 20 °C. This indicates that that the surface evaporation of Ca was faster than that of Mg. The Mg lubricant migrated to the PP surface relatively weakly during thermal aging at low temperatures. Therefore, with Mg, surface blooming was inhibited more than with Ca.

The contact angles changed only slightly with temperature and, overall, could be considered constant. This phenomenon might be attributed to the contact angle of the pure lubricant, although it was observed that surface migration led to changes in the contact angle. The PP/lubricant composite samples changed color at the surface, and the formation of water spots and whitening were observed. The contact angle values measured after migration due to thermal aging were 101° and 99° for Mg, and Ca, respectively. The complex changes in the contact angle of water, described above, were interpreted thermodynamically with reference to the surface free energy of the solids. The surface free energy is assumed to consist of two parts, a polar component, which accounts for the dipole–dipole and H bonding interactions, and a dispersive component, which accounts for the London-type (van der Waals) forces. The surface free energies were calculated by applying the least squares method to Equation (2) and using the contact angles and surface energies of water and diiodomethane. Figure 3c1,c2 shows the results of these calculations. After thermal aging at 90 °C, all the samples showed a decreasing trend in the surface energy, but the Mg lubricant increased the surface energy at 90 °C. This might be due to the surface evaporation of the lubricants at high temperatures. We assumed that the polar functional groups introduced into the polymer surface by the aging treatment migrated from the inner bulk of the polymer to the surface. High surface energy values were obtained for samples treated at 20–50 °C in the case of PP/Ca composites. These results indicate that a higher surface energy leads to a more significant movement of the lubricant to the PP surface.

### 3.2. Surface Free Energies of PP and Lubricants

**Figure 4:**
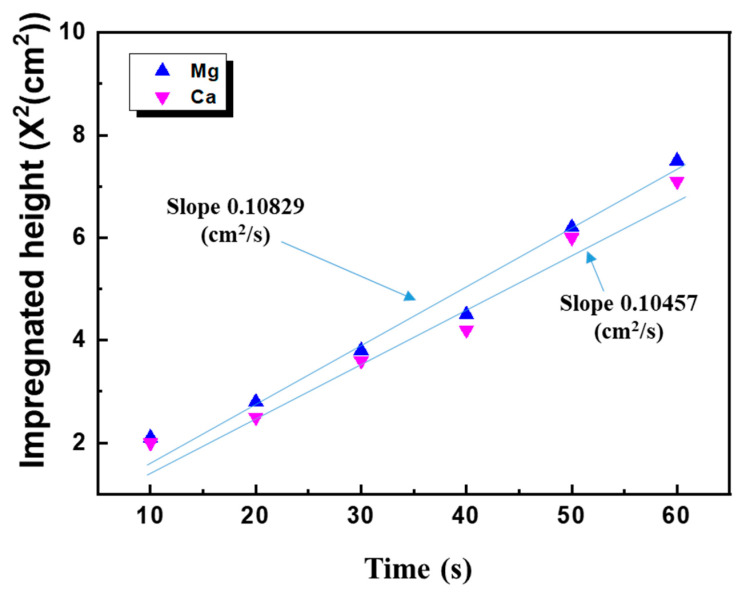
Penetrated distance squared of n-hexane infiltrating lubricant as a function of time.

The surface free energy of PP was evaluated to study the adhesion of the PP/lubricant composites. The surface free energy parameters calculated using Equation (2) are listed in Table 3. Figure 4 shows the penetrated distances squared X^2^ as a function of time, and t for the two lubricants wicked with n-hexane. The curve of X^2^ as a function of t (Equation (3)) traverses precisely through the origin. However, in practice, this does not occur owing to various hydrodynamic disturbances at during immersion. Therefore, we plotted h^2^ as a function of t, and the results are shown in Figure 4. The capillary effective radius, R, can be obtained by calculating the slopes of the curves in Figure 4. The *γ_S_^LW^* values of the lubricant were obtained using a diiodomethane infiltrating lubricant, after which the Lewis acid-alkali forces of the fillers can be calculated using distilled water and formamide. The results are shown in Table 3.

From the Table 4, it can be seen that there is a large difference between the surface free energies of different lubricants. Generally, the surface free energy increases with decreasing particle size due to the presence of more high-energy sites [17]. This is attributed to the differences in their Lewis acid-alkali forces. However, the van der Waals forces of the different lubricants are nearly the same. The Mg lubricant exhibited the highest surface free energy, whereas the Ca lubricant exhibited the smallest Lewis acid-alkali force.

### 3.3. Work of Adhesion of PP/Lubricant Composites

There are two types of migration according to thermal and humidity damage forms in PP: surface migration, i.e., evaporation due to low interactions between PP and the lubricant, and surface blooming, i.e., degradation of surface-migrated lubricant by thermal and humidity aging. Both surface migration and blooming are related to the surface free energies of PP and the lubricant. The surface energy of a solid at a given temperature determines its adsorption wettability, catalysis, permeation, dyeability, painting, and adhesion. The work of adhesion is the work done on the system when two condensed phases A and B, forming an interface of unit area, are separated reversibly to an infinite distance. According to Wu [15], this can be calculated from the interfacial tension.

The estimated values of the surface tension, dispersion component, and polar component are listed in Table 3 and Table 4. The work of adhesion (*W_AB_*) was determined from the surface tension values using the following equation:

(7) $$W_{AB}=2(\gamma_{A}^{d}\gamma_{B}^{d}{)}^{1/2}+2(\gamma_{A}^{p}\gamma_{B}^{p}{)}^{1/2}$$

where *W_AB_* is the work of adhesion, and *γ_A_* and *γ_B_* are the surface tensions of the two materials in contact.

**Figure 5:**
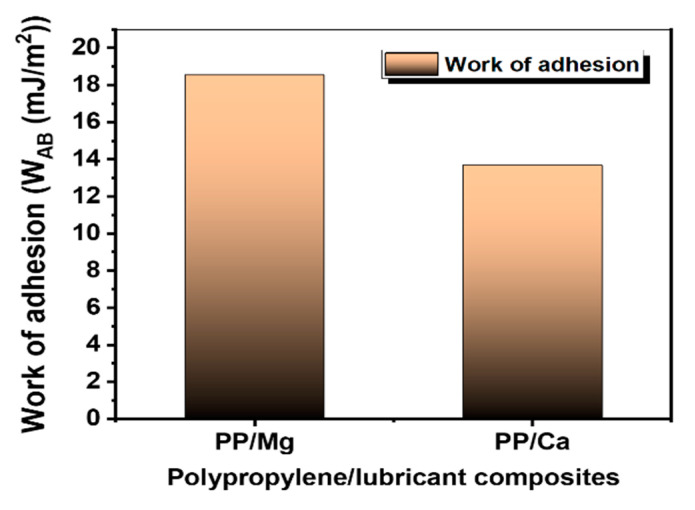
Work of adhesion of polypropylene/lubricant composites.

The values of the work of adhesion of all PP/lubricant composites are shown in Figure 5. From the figure, it can be observed that the work of adhesion of the PP/Mg interface was higher than that of PP/Ca composites. A larger work of adhesion of the interface area lead to a stronger resistance to surface migration. These results support the surface migration rate phenomenon, which shows a more stable behavior in the accelerated aging temperature environment than Ca in the case of Mg, which has a high work of adhesion value with PP.

### 3.4. Characterization of PP/Lubricant Composites

**Figure 6:**
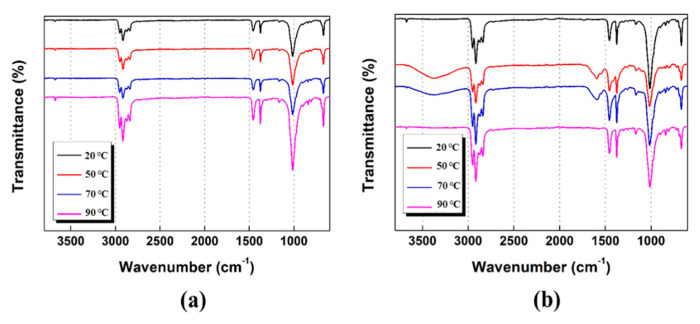
FTIR spectra of polypropylene/lubricant composites aged at different temperatures at a 100% humidity: (**a**) PP/Mg; (**b**) PP/Ca.

Figure 6 shows the ATR-FTIR spectra of the PP/lubricant composites aged at different temperatures and 100% humidity. All the spectra showed similar trends after thermal aging, as chemical modification involves the same functional groups, which can explain the reduction in the carbonyl group and lubricant contents in aged PP/lubricant composites. The characteristic peaks in the ATR-FTIR spectra confirm the surface modification. The peak intensity of the Mg group of magnesium stearate at 2915 and 2855 cm^–1^ increased with increasing temperature (Figure 6a,b). The reason for this variation may be ascribed to the degradation and evaporation occurring during the accelerated aging of the PP/lubricant composites. A sharp peak at ~1600 cm^−1^, which corresponds to the Ca group of calcium stearate, was not observed when the sample was aged at 20 and 90 °C; this is because a slight surface shift occurred at 20 °C and evaporation occurred at 90 °C. On the other hand, at 50–70 °C, the peak intensity of the Ca group on the PP surface increased. This result is different from that obtained when Mg was used as lubricant. Therefore, the peak intensity is affected by the type of lubricant and treatment temperature.

**Figure 7:**
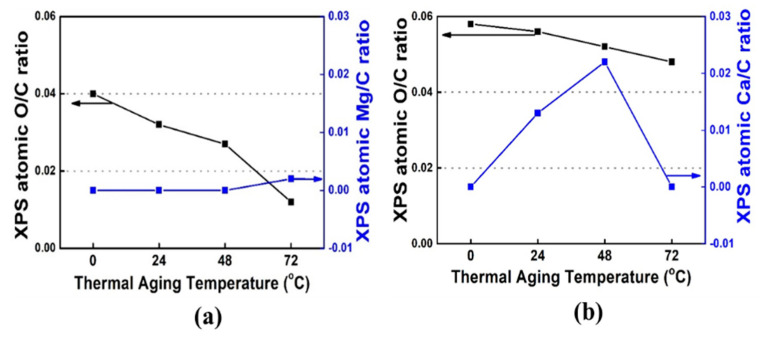
XPS atomic ratio of polypropylene/lubricant composites aged at different temperature conditions under 100% humidity: (**a**) PP/Mg; (**b**) PP/Ca.

**Figure 8:**
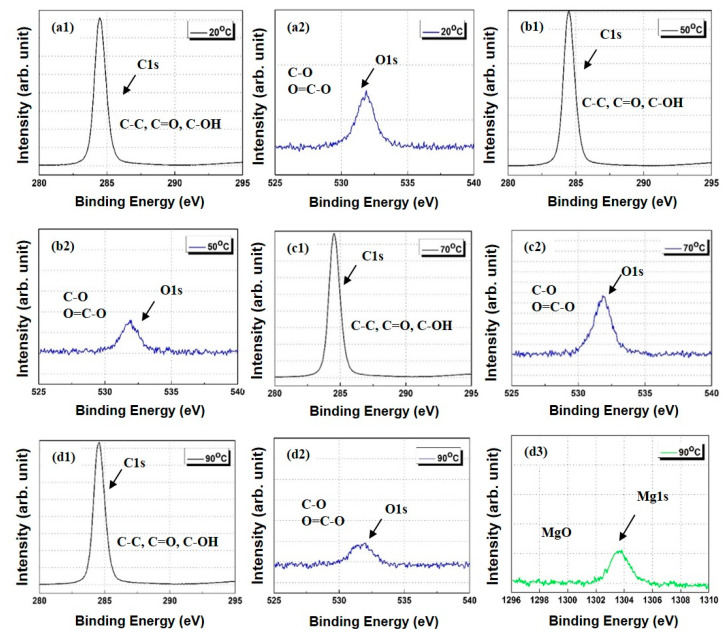
XPS spectra of polypropylene/lubricant (Mg) composites aged at different temperature conditions at 100% humidity: (**a**) C1s and O1s spectra at aging temperature of 20 °C; (**b**) C1s and O1s spectra at aging temperature of 50 °C; (**c**) C1s and O1s spectra at aging temperature of 70 °C; (**d**) C1s, O1s, and Mg1s spectra at an aging temperature of 90 °C.

**Figure 9:**
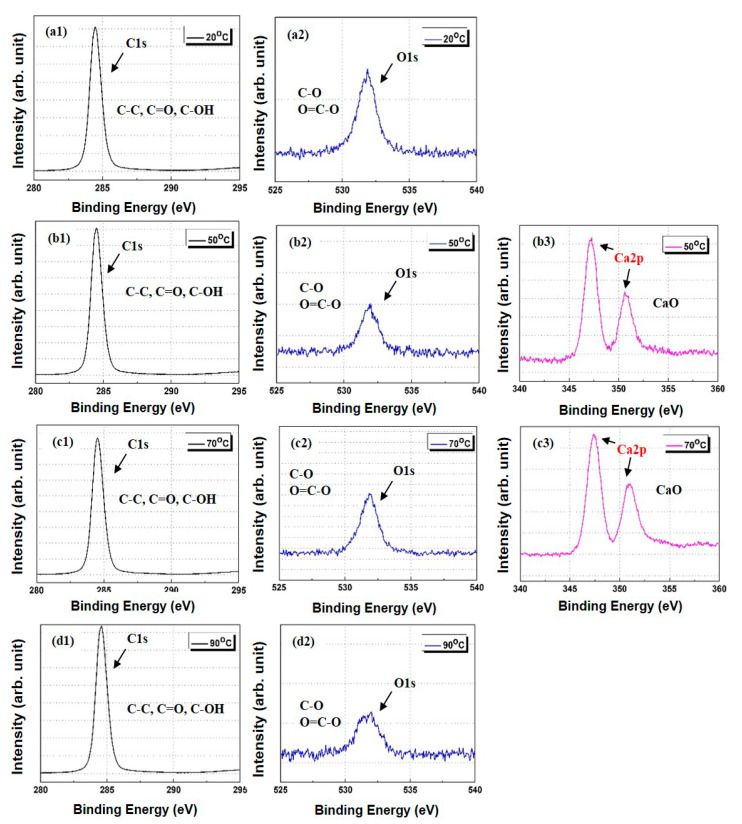
XPS spectra of polypropylene/lubricant (Ca) composites aged at different temperature conditions under 100% humidity: (**a**) C1s and O1s spectra at aging temperature of 20 °C; (**b**) C1s, O1s, and Ca2p spectra at aging temperature of 50 °C; (**c**) C1s, O1s, and Ca_2p_ spectra at aging temperature of 70 °C; and (**d**) C1s and O1s spectra at aging temperature of 90 °C.

An XPS analysis was conducted to validate the FTIR results. The XPS patterns of the PP/lubricant composites exhibited a decrease in the O/C atomic ratio after thermal aging, which indicates a decrease in the carbonyl group content at the PP/lubricant composite surfaces (Figure 7a,b). The hydroxyl and carbonyl groups are responsible for the hydrophilicity of the PP/lubricant composites. Figure 8 and Figure 9 show the carbon (C_1s_) and oxygen (O_1s_) spectra of the PP/lubricant composites, respectively, after thermal aging. The spectra of the aged PP/lubricant composites show an additional peak in the Ca_2p_ spectra at 347 and 351 eV and Mg_1s_ spectra at 1304 eV corresponding to magnesium stearate and calcium stearate, respectively. The oxygen/carbon ratio for the aged PP/lubricant composites at 20 °C was higher than that of the aged PP/lubricant composites at 90 °C. This was mainly due to the surface migration and evaporation of the hydroxyl and carbonyl groups. In the case of a lubricant (Mg) with a high work of adhesion between PP and the lubricant, a binding energy peak corresponding to Mg was observed at an aging temperature of 90 °C, whereas Ca was observed at a lower aging temperature of 50 °C. However, Ca was evaporated by surface migration at an aging temperature of 90 °C due to the low work of adhesion with PP, resulting in the disappearance of the Ca binding energy peak. The resulting XPS surface element concentrations of the PP/lubricant composites are listed in Supplementary Materials Table S1.

### 3.5. Surface Morphology of PP/Lubricant Composites

**Figure 10:**
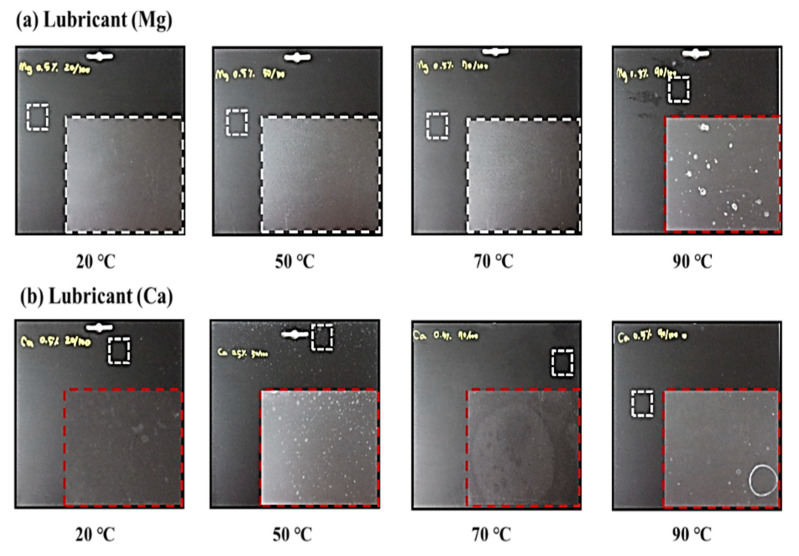
Close-up distal images of polypropylene/lubricants composites thermally aged at different temperatures under 100% humidity: (**a**) PP/Mg; (**b**) PP/Ca.

**Figure 11:**
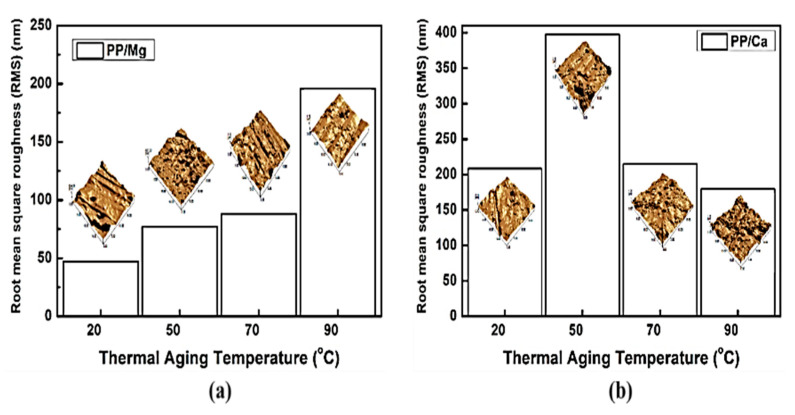
AFM images of polypropylene/lubricants composites thermally aged at different temperatures under 100% humidity: (**a**) PP/Mg; (**b**) PP/Ca.

Figure 10a,b shows close-up digital images of the PP/lubricant composites subjected to different thermal aging conditions at 100% humidity. In the PP composites manufactured with different lubricants Mg or Ca (Figure 10a,b), migration phenomena related to whitening and blooming (PP/Mg composites) were observed at aging temperatures of 90 °C (Figure 10a). Migration of PP/Mg composites was clearly indicated by the whitening and blooming due to the strong work of adhesion of Mg with PP at a high aging temperature of 90 °C. On the other hand, migration was observed at 20 °C in the PP/Ca composites. The PP/Ca composites with a low work of adhesion exhibited rapid migration at lower temperatures. This result was found to be dependent on the value of the work of adhesion. Figure 11a, b shows AFM images of the PP/lubricant composites with different thermal aging treatments at a 100% humidity. The composites that did not exhibit surface migration had surfaces smoother and more homogeneous than those that exhibited surface migration. The RMS values and average roughness values (Ra) of all PP/lubricant composites are listed in Supplementary Materials Table S2. We concluded that the RMS and Ra values were significantly decreased at the highest aging temperature except in the case of the PP/Mg composites. Obviously, only non-migration and surface evaporation of lubricants decreased the surface roughness, while the surface migration (whitening, blooming, and white spots) of the lubricants increased the surface roughness of PP/lubricant composites by some extent.

## 4. Conclusions

The migration of different lubricants in polypropylene composites and their effect on the surface characteristics were studied. It was concluded that the PP composites manufactured using Mg as the lubricant exhibited stable migration when accelerated aging was carried out at 20–90 °C and 100% humidity for 72 h; with these parameters, a slower migration phenomenon was observed with the Mg lubricant than with the Ca lubricant. These observations were confirmed by ATR-FTIR, XPS, AFM, and close-up digital imaging analyses. In addition, it was revealed that the stability of the surface migration of lubricants can be improved by increasing the work of adhesion between PP and the lubricants, which eventually contributes to the quality enhancement of PP/lubricant composites.

## Figures and Tables

**Table 1:** Values of the total surface free energy(*γ_l_*), dispersive component(*γ_l_^d^*), and polar component (*γ_l_^p^*) of test liquid for PP/lubricant composites.

Liquids	Surface Free Energy Components (mJ/m^2^)		
	*γ_l_*	*γ_l_^d^*	*γ_l_^p^*
Distilled water	72.8	21.8	51.0
Diiodomethane	50.8	50.4	0.38

**Table 2:** Surface free energy(*γ_L_*), Lifshitz-van der Waals force(*γ_L_^LW^*), Lewis acid force(*γ_L_^+^*), Lewis alkali force(*γ_L_^−^*), and viscosity(*η*) of test liquid for polypropylene.

Liquids	*γ_L_* (mJ/m^2^)	*γ_L_^LW^* (mJ/m^2^)	*γ_L_^+^* (mJ/m^2^)	*γ_L_^−^* (mJ/m^2^)	*η* (mPa⋅s)
Distilled water	72.8	21.8	25.5	25.5	1.002
Diiodomethane	50.8	50.8	0	0	2.762
Formamide	58.0	39.0	2.28	39.6	4.550
Toluene	28.3	28.3	0	2.7	0.59
Chloroform	27.3	27.3	3.8	0	0.786

**Table 3:** Surface free energy components of polypropylene.

	Surface Free Energy *γ_a_* (mJ/m^2^)	Dispersion Force *γ_a_*^d^ (mJ/m^2^)	Polarity Force *γ_a_*^p^ (mJ/m^2^)
Polypropylene	31.88 ± 2.1	31.76 ± 3.2	0.12 ± 2.7

**Table 4:** Surface free energy(*γ_s_*), Lifshitz-van der Waals force(*γ_s_^LW^*), Lewis acid-alkali force(*γ_s_^AB^*), Lewis acid force(*γ_s_^+^*), and Lewis alkali force(*γ_s_^−^*) components of lubricant.

Lubricant	*γ_s_* (mJ/m^2^)	*γ_s_^LW^* (mJ/m^2^)	*γ_s_^AB^* (mJ/m^2^)	*γ_s_^+^* (mJ/m^2^)	*γ_s_^−^* (mJ/m^2^)
Mg	7.60 ± 0.4	5.34 ± 0.1	2.26 ± 0.3	0.25 ± 0.2	2.01 ± 0.1
Ca	6.49 ± 0.6	4.84 ± 0.1	1.65 ± 0.5	0.31 ± 0.3	1.34 ± 0.2

## Data Availability

The data presented in this study are available on request from the corresponding author.

## Supplementary Materials

The following are available online at https://www.mdpi.com/article/10.3390/polym13111723/s1, Table S1: XPS surface element analysis data of polypropylene/lubricants composites aged at different temperatures and 100% humidity, Table S2: Roughness parameters of thermal aged polypropylene/lubricant composites by AFM on images of 20 um × 20 um.
