# Supplementary material for: Migration Behavior of Lubricants in Polypropylene Composites under Accelerated Thermal Aging
Source: Polymers (Basel). 2021 May 25;13(11):1723. doi: 10.3390/polym13111723 (PMC8197380; doi:10.3390/polym13111723)
Supplement: Supplementary file 1 [file polymers-13-01723-s001.zip › polymers-1203174 SI.pdf]

Supporting Information

Migration Behavior of Lubricants in Polypropylene Composites under Accelerated Thermal Aging

Mun Gyu Bak [1], Jong Sung Won [2], Seon Woong Koo [3], Arom Oh [1], Han Ki Lee [1], Dae-Sik Kim [1] and Seung Goo Lee [3,*]

[1] Department of Plastic Materials Research Team, Automotive Research & Development, Hyundai Motor Group, Hwaseong-si 445-010, Korea; mg_bak@hyundai.com (M.G.B.); arom@hyundai.com (A.O.); HK_Lee@hyundai.com (H.K.L.); kimds@hyundai.com (D.-S.K.)
[2] Robert Frederick Smith School of Chemical and Biomolecular Engineering, Cornell University, Ithaca, NY 14853, USA; jw2636@cornell.edu
[3] Department of Advanced Organic Materials & Textile Engineering, Chungnam National University, Daejeon 34134, Korea; muzicle@torayamk.com
* Correspondence: lsgoo@cnu.ac.kr

Table S1. XPS surface element analysis data of polypropylene/lubricants composites aged at different temperatures and 100% humidity.

**Mg**	**Chemical Composition (%)**			**Atomic Ratio**		**Binding Energy (eV)**
	**C**	**O**	**Mg**	**O/C**	**Mg/C**	C-C (285)
**20 °C**	96.52	3.48	-	0.040	-	C=O (287.6)
**50 °C**	96.85	3.15	-	0.032	-	C-OH (286.1)
**70 °C**	97.31	2.69	-	0.027	-	C-O (532)
**90 °C**	98.65	1.19	0.16	0.012	0.002	O=C-O (534)<br>MgO (1304)
**Ca**	**Chemical Composition (%)**			**Atomic Ratio**		**Binding Energy (eV)**
	**C**	**O**	**Ca**	**O/C**	**Ca/C**	C-C (285)
**20 °C**	94.53	5.47	-	0.058	-	C=O (287.6)
**50 °C**	93.51	5.30	1.19	0.056	0.013	C-OH (286.1)
**70 °C**	92.6	5.35	2.05	0.052	0.022	C-O (532)
**90 °C**	95.41	4.59	-	0.048	-	O=C-O (534)<br>CaO (347, 351)

Table S2. Roughness parameters of thermal aged polypropylene/lubricant composites by AFM on images of 20 um × 20 um.

Composites	Aging temperature (°C)	Root mean square roughness, RMS (nm)	Mean roughness, Ra (nm)
Polypropylene/Mg	20	47.11	41.04
	50	77.01	59.50
	70	87.76	74.51
	90	195.56	165.62
Polypropylene/Ca	20	208.45	148.25
	50	397.34	314.55
	70	214.73	181.57
	90	179.36	148.01
